# FLO11, a Developmental Gene Conferring Impressive Adaptive Plasticity to the Yeast *Saccharomyces cerevisiae*

**DOI:** 10.3390/pathogens10111509

**Published:** 2021-11-19

**Authors:** Clara Bouyx, Marion Schiavone, Jean Marie François

**Affiliations:** 1 Toulouse Biotechnology Institute, UMR-CNRS 5504 and INRA 792, 31077 Toulouse, France; bouyx@insa-toulouse.fr (C.B.); mschiavone@lallemand.com (M.S.); 2 Lallemand SA, 10, Rue des Briquetiers, 31702 Blagnac, France

**Keywords:** *Saccharomyces cerevisiae*, cell wall, flocculins, adhesins, *FLO11*, phenotypic variation, adaptive plasticity

## Abstract

The yeast *Saccharomyces cerevisiae* has a remarkable ability to adapt its lifestyle to fluctuating or hostile environmental conditions. This adaptation most often involves morphological changes such as pseudofilaments, biofilm formation, or cell aggregation in the form of flocs. A prerequisite for these phenotypic changes is the ability to self-adhere and to adhere to abiotic surfaces. This ability is conferred by specialized surface proteins called flocculins, which are encoded by the *FLO* genes family in this yeast species. This mini-review focuses on the flocculin encoded by *FLO11*, which differs significantly from other flocculins in domain sequence and mode of genetic and epigenetic regulation, giving it an impressive plasticity that enables yeast cells to swiftly adapt to hostile environments or into new ecological niches. Furthermore, the common features of Flo11p with those of adhesins from pathogenic yeasts make *FLO11* a good model to study the molecular mechanism underlying cell adhesion and biofilm formation, which are part of the initial step leading to fungal infections.

## 1. Introduction

When microbial cells are challenged by unfavorable environmental conditions, evolutionary models predict that natural selection favors genetic changes that give cells an advantage in an adverse condition. For microbes that reproduce asexually and very quickly, random mutations can generate genetic diversity to adapt in fluctuating environments. However, many other strategies are employed by microbial cell populations to generate phenotypic diversity and adapt to their environments. The budding yeast *Saccharomyces cerevisiae* has conceived an original adaptive response that consists of switching from a unicellular to a multicellular lifestyle according to the environmental conditions. Flocculation is among the best-known multicellular growth forms of yeast, which was originally described by Emil Hansen more than 100 years ago [1]. While the classic definition of flocculation is the reversible calcium-dependent aggregation of thousands of vegetative cells into flocs [2,3], works from the Verstrepen group showed that the flocculation is a cooperative protection mechanism that shields cells from stressful environments and protect them against cheaters cells, such as excluding *flo1^-^* negative cells from the flocs [4]. Velum is another lifestyle where yeasts form a buoyant biofilm at the air–liquid interface at the end of an alcoholic fermentation process as an adaptive mechanism to gain access to oxygen in these poor oxygenated but high ethanolic environments [5,6]. Under deleterious nutritional condition, such as nitrogen starvation or glucose depletion, *S. cerevisiae* can trigger a filamentous program in the form of pseudohyphae, which correspond to chains of attached cells that are formed from one another by budding [7,8,9,10]. This morphological change enables cells to penetrate solid substrates and to grow invasively, a phenotype that is commonly monitored by invasion in agar plates [11,12]. The yeast *S. cerevisiae* is also able to form biofilms as mats, which are aggregated cells on a semi-solid agar surfaces exposed to air and may correspond to what they likely experience in rotting fruits [13]. Biofilms can also be formed on abiotic surface such as plastic or polystyrene [14]. These phenotypic behaviors, of which a nice illustration can be found in reference [15], are thus taken as a model to study the molecular basis of biofilm formation, as it is the most common mode of colonization and infection of host cells and tissues by pathogenic yeasts, such as *Candida albicans* [16,17].

A key feature in this multicellular behavior is the original propensity of wild *S. cerevisiae* for cell–cell adhesion and cell–surface adhesion. This prerequisite is brought about by a family of glycosylphosphatidylinositol-anchored cell wall proteins (GPI-CWPs) termed flocculins, which are encoded by *FLO1*, *FLO5*, *FLO9*, *FLO10,* and *FLO11*. Although the *S. cerevisiae* genome is endowed with these five genes encoding flocculins, to which could be added *FIG2* and *AGA1* encoding cell-wall associated surface proteins that also belong to adhesins family but do not display the same sequence architecture as *FLO* encoded flocculins [18], we will concentrate this mini-review on the flocculins encoded by *FLO11*. We will discuss the recent work about the relationship between protein sequence/structure and molecular function, notably in term of cell–cell and cell–substrate adhesion. We will examine what differentiates Flo11p from the other flocculins and how this cell–surface protein is capable of engendering so many varied phenotypic behaviors, leading to consider Flo11p as a masterpiece for yeast adaptation and evolution. Flo11p may be taken as a model for understanding cell–cell and cell–surface adhesion mechanisms that are exploited by pathogenic yeasts to adhere to abiotic surfaces such as catheters and gain access to the internal organs of patients or to serve as a reservoir of drug-resistant infectious cells in the form of biofilms.

## 2. Primary Sequence Analysis Distinguishes Flo11p from the Other Yeast Flocculins

**Figure 1:**
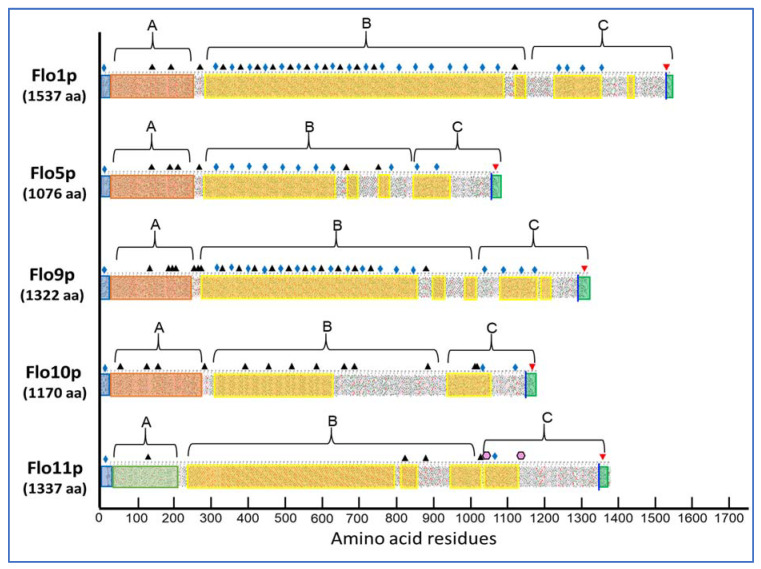
Primary structure analysis of the flocculins encoded by the gene family of the *Saccharomyces cerevisiae* strain S288c as represented by a hydrophobic-cluster analysis (HCA). The secretion signal sequence and the GPI addition signal at the C-terminus are boxed in blue and green, respectively. The vertical blue line denotes the GPI signal cleavage and anchorage to the cell wall β-1,6 glucan. The N-terminal part that corresponds to the A-domain is represented as an orange box for Flo1 to Flo10p, whereas it is boxed in light green to indicate its structural difference with the A domain of the other Flo proteins. Tandem repeats in B-domain that are Ser/Thr rich are represented by yellow boxes. Potential N-glycosylation are marked by black triangles. Sequence with β-aggregation potential of >30% as predicted by TANGO (http://tango.crg.es/) (accessed on 15 October 2021) is marked with a blue diamond and the amyloid-core sequence is marked by pink hexagons.

The *S. cerevisiae* flocculins belong to a large family of fungal glycosylphosphoinositide-linked cell wall proteins (GPI-CWPs). As illustrated in Figure 1, these flocculins, which are encoded by 5 genes, exhibit a modular architecture with an N-terminal domain (A-domain) flanked by a secretory signal sequence, a large middle region (B-domain) containing serine/threonine-rich repeats, and a C-terminal part at which a remnant of GPI anchor establishes the cross-link between an amino acid of this C-domain and a non-reducing end of β-1,6-glucan, which involves a transamidation and transglycosylation reactions, respectively [19]. The representation of the primary structure of the flocculins by a hydrophobic-cluster analysis (HCA) [20] already points to differences between Flo11p and the other flocculins. First, on a global sequence, the similarity between Flo1p and Flo11p is only 37% whereas it is >90% between Flo1p, Flo5p, and Flo9p [18]. Second, the A-domain that spans about 200–230 amino acids at the N-terminus of the flocculin is very different in sequence and structure between Flo11p and the other flocculins. For Flo1 to Flo10p, this domain encompasses a PA14 lectin domain, which is widely distributed in the Bacteria, Archaea, and Eukarya domain, and harbors a unique Ca^2+^-binding motif D*cis*D. It also contains a so called Flo subdomain that is exposed at the cell surface and bears the carbohydrate binding site and [21,22]. This type of A-domain fully accounts for the dominant Ca^2+^-dependent flocs formation. For further details on the structure of the P14-Flo domain, the reader should consult the recent review on adhesins in yeast [23].

In contrast, the structural analysis of the A-domain of Flo11p has revealed a fibronectin type III-like adhesion domain that does not have any mannose-binding sites [24]. Interestingly, this type of domain is found only in the ascomycetal orders of *Saccharomycetales*, and in the Flo11p of *Komagataella pastoris* (formerly known as *Pichia pastoris* [25]) although sharing only 32% homology with ScFlo11p [26]. Interestingly, this A-domain is present in up to three times in adhesins of the human pathogenic *Candida lusitaniae* and the wood-boring beetle associated fungus, *Spasthasphora passalidarum* [24].

A third significant difference between Flo11p and the other flocculins is found at the large middle region (B-domain) of these proteins. Albeit the primary sequence in this B-domain consists of tandem repeats, this analysis eventually splits the flocculins into two categories. The first one that comprises Flo1p, Flo5p, and Flo9p is characterized by a large number of short sequences of 5 to 7 amino acids especially rich in β-branched amino acids Val, Ile, and Thr with a β-aggregation potential > 30% as predicted by TANGO predictor software (http://tango.crg.es/) (accessed on 15 October 2021) More than 50% of these β-aggregation prone sequences have the consensus T(V/I)IVI and they are all present in the 45-residue repeats of these flocculins. It is considered that high β-aggregation potential sequences provide amyloid fiber properties to these proteins [27]. Ramsook et al. [28] indeed showed that a synthetic peptide containing a Flo1p TANGO positive sequence forms amyloid fibers in vitro. The second category encompasses Flo10p and Flo11p, which exhibit less β-aggregation complexity within the B-domain. However, Flo11p possesses at its C-terminus two β-aggregation sequences that nicely match the amyloid-core sequence VVSTTV or VTTAVT, which is supported by the finding that this protein is also able to aggregate into amyloid fibers in vitro [28,29] and in vivo ([30] see below).

## 3. Relationship between Sequence/Structure and Role in the Physiological Function of Flo11p

### 3.1. Strain Phenotypes Are Shaped by the Plasticity of the FLO11-Encoded Protein

The gene encoding Flo11p was originally isolated and characterized independently by two research groups from a *Saccharomyces cerevisiae* var. *diastaticus* [31,32], which is a yeast able to grow on starch due to the production of a glucoamylase encoded by any one of the members of the *STA* genes family [33]. Two seminal properties of this flocculin were already disclosed in these works, which were the requirement of Flo11p for invasive and pseudohyphal growth under carbon or nitrogen limitation and its involvement in flocculation. Subsequent work revealed other phenotypes, including the formation of velum, which is a biofilm of yeast cells floating on the surface of a wine barrel [5,34,35], and mats, which correspond to a floral-like biofilm that expands over a wet, semi-solid surface by sliding motility [14]. Adhesion of yeast cells on solid surfaces such as plastic or polystyrene was reported as a property elicited by Flo11p [12]. Additionally, Flo11p was shown to be implicated in colony morphologies exhibited by different yeast strains growing on solid media in the presence of various carbon sources [36]. More recently, we showed that Flo11 molecules could cluster together to form adhesion nanodomains on the cell surface, a property that is dependent on a threshold number of amyloid-β-aggregation prone sequences in the Flo11p ([37]; discussed below). This wide variety of phenotypes raises the question of whether they can be expressed collectively in a single yeast strain and which domain(s) of Flo11p is/are responsible for these phenotypes. Works from the Hyman [38] and Verstrepen teams [39] have in part provided some answers to the first question. They both used the laboratory strain S288c in which *FLO* genes are not expressed because of a nonsense mutation in the major transcriptional activator encoded by *FLO8* [40]. While one group integrated individual *FLO* genes under the strong *TEF* promoter, the other expressed *FLO11* in a high copy plasmid under the galactose inducible *GAL1* promoter. These works led to two major findings regarding differences between Flo11p and the other flocculins. On the one hand, only Flo11p has the ability to trigger strong invasive growth in a semi-solid environment, and thus it is essential in mats formation [41]. However, this property only occurs if the *FLO8* gene is functional, suggesting that while the absence of *FLO11* prevents the invasion process, the latter requires other factors under the control of *FLO8*. On the other hand, *FLO11* is unable to induce a flocculation phenotype, whether *FLO8* is active or not, although agglutination of 10–30 cells can be observed under an optical microscope in a strain that expresses only this gene, indicating that Flo11p promotes cell–cell interactions but not to the extent that it induces aggregation of thousands of cells leading to flocculation. Since this result was at variance to Flo11p-mediated flocculation of the *S. cerevisiae* var. *diastaticus* strain [42], it was argued that strain-specific difference in the Flo11p phenotype may result from significant sequence differences in the *FLO11* alleles, rather than quantitative differences in *FLO11* expression [43]. This assertion was further supported by the fact that laboratory strain S288c is unable to exhibit a flor phenotype or to produce nanodomains on its cell surface even upon dramatic overexpression of *FLO11* [37]. In conclusion, these results highlight the importance of the sequence/structure of the Flo11 protein in the expression of these phenotypes.

### 3.2. Role of A, B, and C-Domains in the Physiological Function of Flo11p

As exposed in Figure 1, the Flo11p presents a modular architecture in three domains or regions whose structure–function began to emerge from recent works [24,37,43,44,45]. The N-terminal domain of Flo11p (A-domain also referred as N-Flo11p) of the S288c strain from aa 22 to 207 was purified and its crystal structure solved at 0.89 Å resolution. This N-Flo11p was shown to be composed of three subdomains: a hydrophobic apical region, a β-sandwich of fibronectin type III domain, and a neck domain [24]. The N-Flo11p can be O-glycosylated but not N-glycosylated; although, there is a predicted N-glycosylated site (Figure 1) and it did not harbor any carbohydrate binding site. The relevant property of the N-Flo11p is to show a homophilic Flo1p interaction. This interaction was nicely demonstrated in vitro using surface plasmon resonance analysis [46] and in vivo using mutants that express Flo11p defective of the N-terminus by single-cell force spectroscopy experiments [45]. Cells of these mutants do not interact each other anymore and they also have lost the invasive growth phenotype [24]. More remarkably, this N-Flo11p confers kin discrimination at the species and subspecies level, accounting for social behavior of yeast by allowing aggregation between single cells expressing the same Flo11p and excluding those that do not express Flo11p or a different alleles of *FLO11* as well as cells from different yeast species that express a paralog of Sc*FLO11* [45]. This homophilic interaction depends on evolutionary conserved aromatic amino acids residues, which form two bands that are present at the apical and the neck region of the N-Flo11p [24]. How different N-Flo11p variants can discriminate between homotypic (kin) and heterotypic (non-kin) interactions is still unclear. Nonetheless, this property makes *FLO11* a member of the green beard genes, which confers cooperation between cells that carry the same allele [47]. These exquisite structural details of the N-terminal of Flo11 and their role in adhesion still leave unexplained why Flo11p from *S. cerevisiae* var. *diastaticus* causes flocculation, mediates adherence to plastic, but does not elicit agar invasion [42], even though this protein shows homotypic interaction [29]. Barua et al. [43] compared primary sequence of Flo11p from this strain with that of S288c and Σ1278b. Apart a 15-amino acid insertion in the N-terminal of the Flo11p of Σ1278b, which is otherwise exclusively found in the ‘sake lineage’ of the *S. cerevisiae* strain [48] and confers a higher adhesion force between cells [45], no other difference could be noticed between the N-terminus of these three Flo11p. This could suggest that another genetic component whose function is dependent on the presence of Flo11p is implicated in the phenotypes shown by *S. cerevisiae* var. *diastaticus.*

The Flo11p harbors at its carboxyl terminal a typical GPI-attachment site “GAANIKVLGNFMWLLLALPVVF” that is composed of the ω-site (G) at position 1346 followed by a hydrophobic-like sequence termed pro-peptide (aa 1347 to 1376) [49]. This attachment signal is cleaved off and replaced by a preformed GPI-anchor in the endoplasmic reticulum, which enables trafficking of the modified protein via the classical secretory pathway to end up at the plasma membrane as GPI-PMPs [26]. A still unresolved issue is how some of these GPI-PMPs are sorted into the cell walls to be covalently linked to β-1,6-glucan, which involves a transglycosylation mediated by a Dfg5 enzyme [50,51]. Based on an in silico analysis of 51 protein sequences with putative GPI attachment sites, Caro et al. [49] proposed that the presence of two basic amino acids upstream of the ω site at the C-terminus of these GPI-anchored proteins would in most cases dictate retention at the membrane, whereas GPI-PMPs that do not have these motifs, such as Flo11p, are transferred to the cell wall. Another model is that of Vogt et al. [51] who propose that it is the ethanolamine phosphate (EtN-P) modifications on the central GPI glycan that partly dictate the transfer of GPI-PMPs to the cell wall, with the enzyme Dfg5 acting as a decoder of the presence of the EtN-P modification on these GPI-glycans. Whatever the model, the destination of a GPI-anchored protein to the membrane or to the cell wall is not absolute. Accordingly, it was reported that Flo11 protein defective in the C-terminus can be secreted into the culture medium [29], whereas our recent immunofluorescence experiments showed that such a Flo11p variant was still localized at the cell surface.

These results indicate that part of Flo11p could be weakly retained in the cell wall by non-covalent bonding mainly by hydrogen bonding and S-S bridges [52]. Hot SDS-β-mercaptoethanol treatment of the yeast cell wall should be tested to validate this hypothesis [53]. Moreover, the finding that Flo11p can shed from the cells may also agree with the fact that not all Flo11 proteins are covalently attached to the β-1,6-glucan Moreover, this shedding involves a cleavage within the N-terminus of Flo11p by the protease Kex2p [54]. Nonetheless, the major consequence of losing the C-domain of Flo11p is that a yeast strain expressing this variant is no longer able to grow invasively in a semi-solid medium and to form mats, and same effects that were found in a *kex2* mutant [37,54]. The loss of these phenotypes can be explained by the inability of the cells to be retained on the agar plate after washing since this Flo11p variant is no longer retained covalently on the cell wall.

Although there are less data on the structure and function of the central part of the *S. cerevisiae* Flo11p protein, referred as the B-domain (Figure 1), than that of the N- and C-terminus, several studies attest to its strategic importance in the function of Flo11p. The B-domain contains several tandem repeated Ser/Thr sequences predicted to be O- and N-glycosylated, but they are comparatively less numerous than Flo1p, which could explain the higher hydrophobicity character of the latter [55]. This B-domain is also critically important for the velum phenotype of the *flor* strains of *S. cerevisiae* as reported by Fidalgo et al. [5]. These authors found that the Flo11p of the flor strain 133d had twice as many tandem repeats as its counterpart in the laboratory strain S288c and that this higher number of repeats was accompanied by increased cell hydrophobicity, suggesting that this gain in hydrophobicity was critical to the floatability property of this strain. The same argument of cell surface hydrophobicity to elicit this buoyancy property was proposed by Zara et al. [35]. In addition, these authors showed a good correlation between length of the B-domain and potency of making this type of biofilm. This conclusion was completed by Fidalgo et al. [56] who indicated that in addition to the length variation in repeats, changes in the order and/or proportion of the different repeats in Flo11p may be another factor contributing to the buoyant biofilm as well as to other phenotypes including adherence to plastic and invasion in agar. Our recent work carried out with a Flo11p from an industrial strain L69 led to a similar conclusion [37]. Finally, the N-glycosylation status of the B-domain may be another source of phenotypic variation brought about by Flo11p. Using a conditional *pmi40-101* mutant, which is compromised in the early stage of glycosylation due to the loss of phosphomannose isomerase activity at the restrictive temperature, Meem and Cullen [57] showed a significant reduction of invasive growth, mat formation, and pseudohyphal development. It would be worth to verify whether velum formation is also abolished in flor strain defective in this process.

### 3.3. Dual Role of the Amyloid-Forming Sequence in Flo11p-Dependent Cell–Cell Interactions

**Figure 2:**
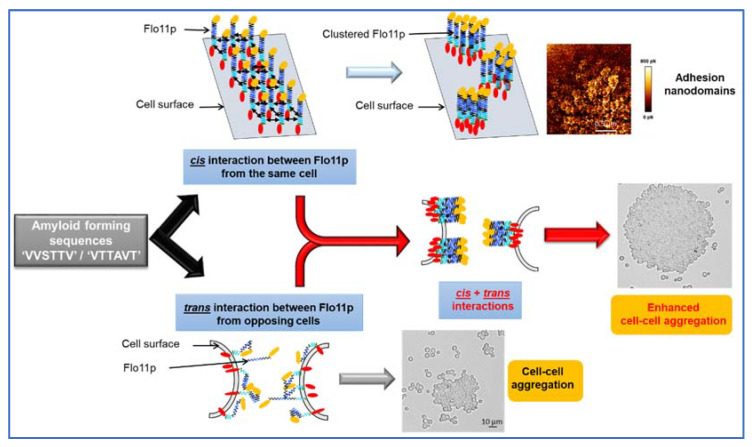
Model by which amyloid-β-aggregation sequences can contribute to nanodomains by *cis*-interaction of Flo11p molecules and cell–cell aggregation by *trans*-interaction of Flo11p of opposing cells.

Fungal adhesins, including the *S. cerevisiae* flocculins have several β-aggregation-prone sequences (see Table 1) that consist mostly of aliphatic β-branched amino acids, which have a high propensity to form β-aggregates. This β-aggregation of proteins increases their local concentration and consequently increases the avidity of binding [58,59]. Physically, the formation of these clusters can be elicited by shear force, such as vortex mixing, laminar flow, or stretching in atomic force microscopy (AFM) [60,61,62]. Moreover, they can be visualized as high avidity adhesins patches termed nanodomains by AFM [62,63]. In the pathogenic yeast, *Candida albicans*, this phenomenon has received a lot of attention because cell surface adhesins control essential processes of adhesion, colonization, and biofilm formation on host tissues and indwelling medical catheters [64]. Work from Lipke and colleagues have shown that these amyloid-core sequences are needed for clustering adhesin molecules in *cis* on the cell surface, but they also mediate cell–cell interaction in *trans* through these cross-β bonds [65,66]. The Flo11p from the laboratory strains S288c and Σ1278b has two typical amyloid core sequences (VVSTTV/VTTAVT) at the boundary between the middle region and the C-terminal domain (see Figure 1), which may likely explain that a soluble version of this protein can assemble into amyloid fibers in vitro [28,29]. In spite of in vitro data and in vivo experiments showing increased cell–cell aggregation upon hydrodynamic shear that can be antagonized by amyloidophilic perturbants [28,30,61], the finding of amyloid-dependent formation of nanodomains and their role in cell–cell adhesion have not been directly demonstrated in the yeast *S. cerevisiae*, until we discovered the formation of abundant patches on the cell surface of an industrial wine yeast L69 strain under the contact of an AFM bare tip [67]. We demonstrated that these patches corresponded to adhesion nanodomains formed by the clustering of Flo11p, which exhibited nanomechanical properties similar to those formed by nanodomains of *Candida albicans* adhesins [63]. The formation of nanodomains on the cell surface of the L69 strain and not on that of the laboratory strain S288c even after overexpression of its endogenous Flo11p was explained by a duplication of a short 100 amino acids sequence near the C-terminal of Flo11p of the industrial strain, which provided two additional amyloid-core sequences (VVSTTV). This data argued that a threshold number of these short β-aggregation prone sequences is necessary for effective nanodomains production under shear force [37]. Moreover, this work provided evidence that amyloid-core sequences contribute to *trans*-interactions between Flo11p of opposing cells, and hence supported the model of Lipke and colleagues for a dual role of amyloid β-sheet interactions; that is, in the formation of clusters of Flo11p on the cell surface (*cis*-interaction) and in homophilic bonding between Flo11p of opposing cells (*trans*-interactions) [66,68]. Taking into account these data and that Flo11p can connect cells by forming an extracellular matrix that involves co-aligned Flo11 fibers as suggested from ultrastructure analysis by electron microscopy [24], we propose the model depicted in Figure 2 in which nanodomains resulting from *cis*-interactions of Flo11p on the cell surface may enhance the homophilic interactions between Flo11 proteins of opposing cells, strengthening henceforth cell–cell interactions.

## 4. Intragenic Repeats Combined with Epigenetic and Conventional Genetic Regulation of *FLO11* Confers an Impressive Evolutionary Plasticity to *S. cerevisiae* to Exploit New Niches and Resources

Although the common criteria of all *FLO11*-dependent phenotypes are the ability of cells to adhere to each other, this broad phenotypic repertoire indicates that this gene is endowed with a unique plasticity that allows yeast to rapidly adapt to hostile environment or to exploit new niches. This plasticity is brought about by three unique features that characterize the *FLO11* gene, which are (i) intragenic repeats in the DNA sequence of *FLO11*, (ii) genetic regulation by nutrients and pheromones signaling pathways, and (iii) epigenetic control. The repetitive nature of highly similar DNA sequences within a gene is the driving force for the generation of new alleles with reduced or expanded repeats units, resulting from frequent slippage and/or recombination events during replication of DNA. Expression of these new alleles can lead to phenotypic changes. These original finding that expansion or contraction of intragenic repeats results in phenotypic variability was shown with *FLO1* encoding a flocculin responsible for flocculation phenotype [69]. These authors reported a seemingly linear relationship between flocculation potency and number of tandem repeats. As reported in Table 2, the number of repeat units, as well as their nature, are quite variable among the *FLO11* of these four yeast strains, which may likely explain why only strain 133d exhibits the so called velum phenotype [5]. Moreover, it was shown that this velum phenotype was not solely dependent on the number of tandem repeats but also on the nature of repetitive units [56]. In addition, these authors demonstrated that a reduction of intragenic repeats in *FLO11* can occur rapidly under neutral (YPD medium) conditions, but not under selective conditions under which this phenotype is needed (i.e., in the wine medium). Indeed, after five passages on YPD, a large fraction of the yeast population contained shorter *FLO11* that have lost several repeats, and consequently the cells expressing these alleles had also lost most of their *FLO11*-phenotype including buoyancy, invasive growth, and adhesion to plastic.

**Figure 3:**
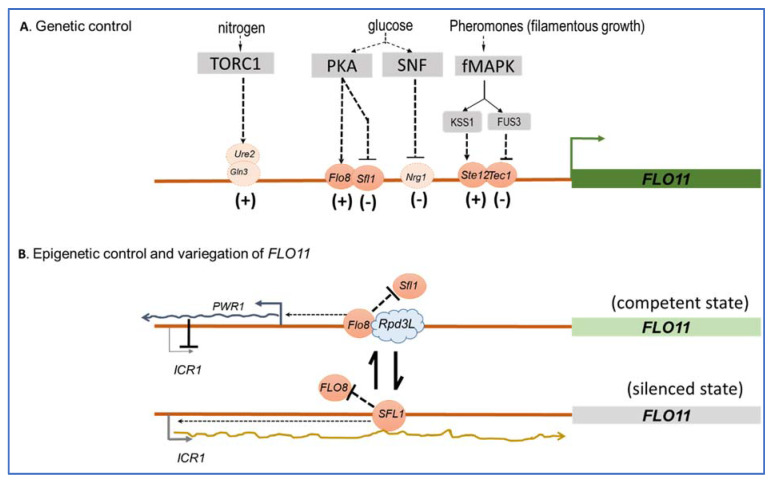
Simplified scheme of the genetic and epigenetic control of the *S. cerevisiae FLO11*. In (**A**) is reported the main signaling pathways with their cognate transcription factors that activate or repress *FLO11*. In (**B**) is illustrated the epigenetic control and transcriptional variegation of *FLO11* that is dependent on the competitive binding of Sfl1 and Flo8 *trans* factor at overlapping binding domain located at −1200 bp from the *FLO11* start codon. This competition also involves the toggling between two nRNAs encoded by *ICR1* and *PWR1*. This ncRNA circuitry leads to the production of a competent (*Flo8*-binding) or a silenced (Sfl1-binding) state that accounts for the variegation of FLO11 in a cell population. Arrows indicate positive regulation and inhibition is shown by bars. Transcription factors that have a positive or negative effects on transcription are indicated by (+) or (−). See text for further details on the mechanism and references [15,82,83] for additional description of *FLO11* genetic and epigenetic regulation.

The *FLO11* promoter spans more than 3 kb to which several transcriptional factors are conveyed that either activate or repress the expression of this gene in response to several environmental clues, which mainly include glucose depletion and nitrogen starvation (Figure 3), but also pH or the quorum-sensing-like signal tryptophol through signaling pathways. The cAMP-PKA and the fMAPK (or pheromone-dependent-MAPK) are the main signaling pathways that regulate *FLO11*-dependent adhesion, invasion, and filamentation in response to nutrient limitation/depletion in vegetative cells [70,71]. The former stimulates *FLO11* expression by activating the transcription factor Flo8p and inhibiting the repressor Sfl1p, whereas Tec1/Ste12p is the transcription factor through which the fMAPK upregulates *FLO11* (see [15] and references therein). It is interesting to notice that these two signaling pathways can compensate each other, such as the loss of *FLO8* results in a lack of *FLO11* expression, which can be overcome by the overexpression of *TEC1* and reciprocally [70,72]. Effects of glucose depletion on *FLO11*-dependent invasive growth in haploid cells was shown to be mediated by the SNF1 kinase pathway [73] through the apparent inhibition of the transcriptional repressor Nrg1 [74], whereas the TORC1 pathway positively regulates filamentation growth in diploid cells through its transcription factors Gln3 and Ure2 in response to nitrogen limitation, in parallel to the cAMP-PKA pathway [75]. The RIM101 cascade, which is known to regulate gene expression under alkaline conditions [76] and is crucial in fungal pathogenicity [77] was found to contribute to mat formation and colony morphology, and this action involves the activation of *FLO11* probably through Nrg1p [36,71]. Finally, several transcription factors including Mss1, Msn1, and Mga1 have been identified as activators of *FLO11* mostly via their capacity to suppress, when overexpressed, mutations in genes in cAMP-PKA, TORC1, or SNF1 signaling pathways (see [15] and reference therein). Therefore, it is unclear to which environmental clues these transcription factors respond and to which signaling pathways they belong. Collectively, this complex transcriptional regulation of *FLO11* indicates that this gene is critically important for the yeast *S. cerevisiae* to readily adapt to environmental fluctuations.

The critical role of *FLO11* in conferring adaptive plasticity of *S. cerevisiae* is further reinforced by the fact that this gene is under epigenetic control [78,79,80,81]. This mode of gene regulation refers to the heritable change in the expression of a gene that is not caused by changes in the underlying gene sequence but involves a slow and random toggle switch between a silenced and a competent promoter state of the gene, resulting in the generation of phenotypic heterogeneity within a clonal population. This strategy can take place without necessarily requiring a change in environmental conditions, but it is precisely a means of anticipating these changes and therefore of being prepared to face them, at least for a small fraction of the cell population. Therefore, this strategy complements very well that of genetic regulation, which involves fast fluctuations between a competent but inactivated, and an activated, state of the gene promoter in response to environmental changes. As they complement each other, the central question is to understand mechanistically how this complementation takes place in the case of the *FLO11* gene. Of note, it was shown that the epigenetic silencing of *FLO11* is dependent on its genomic position and is promoter specific [78]. While determinants of genomic position in the *FLO11* regulation are still unclear, the promoter specificity seems to depend on the antagonistic action of the *trans* repressor Sfl1p and *trans* activator Flo8p, which both bind on overlapping sites located −1200 bp upstream of the *FLO11* start codon. This competitive binding initiates events that contribute to either the stabilization of a silent state (Sfl1p binding) or a competent state (Flo8p binding) in each cell (Figure 3). Taking into account these main *trans* factors and including the fMAPK regulated Tec1/Ste12/Phd1p *trans* factors in the global transcription of *FLO11*, Octavio et al. [80] have proposed a model to account for the kinetic role these different regulators have on the slow promoter fluctuations associated with the epigenetic switch and the fast promoter fluctuations associated with conventional genetic activation. This model considers three class of *FLO11* regulators: class I activators represented by Tec1/Ste12/Phd1p regulate fast promoter fluctuations only; and class II activators, to which Flo8p, Msn1, and Mss1p belong, can convert a silenced state to a competent promoter state. The combination of class II and class I activators both have effects leading to high expression of *FLO11*. An additional regulatory layer on this epigenetic phenomenon, which may explain that the expression of *FLO11* is binary or variegated in a clonal population, i.e., *FLO11* is highly transcribed in some cells whereas it is totally silenced in others [78], involves two non-coding RNAs, themselves regulated by Sfl1p and Flo8p and the chromatin remodeler Rpd3L. These two ncRNAs are *ICR1,* which represses *FLO11* transcription, whereas *PWR1* promotes this transcription. On the other hand, Rpd3L is a chromatin remodeler that can bind in the vicinity of the Flo8p binding site, and probably hinders the access of Sfl1p and/or represses *ICR1*, allowing toggling *FLO11* into a competent state [82]. This additional mechanism contributes to the progression from an unstable or bistable to a stable transcriptional state. Overall, these genetic and epigenetic regulations offer yeast cell flexibility in shaping the distribution of gene expression and phenotype within a population.

## 5. Outlook

In this mini-review, we have illustrated all the phenotypic facets provided by the expression of the *FLO11* encoding flocculins, illustrating the ability of this gene to confer to the yeast *S. cerevisiae* an impressive plasticity to quickly adapt to a fluctuating or hostile environment. More precisely, Flo11p seems to be tailored to allow the yeast to adapt to its ecological niche, as nicely illustrated with the protein expressed in flor strains [5]. This plasticity can largely be explained by the presence of several intragenic repeats of *FLO11*, which from recombination events, leads to the generation of different alleles expressing different Flo11p, and by epigenetic and genetic regulations. This sophisticated regulation leads to phenotypic heterogeneity allowing a fraction of the cell population to test the new environmental conditions while sparing another fraction. To further validate the notion that *FLO11* is a developmental gene enabling yeast to exploit a new ecological niche, a helpful step would be to compare the *FLO11* sequence from a wide variety of *S. cerevisiae* strains from diverse natural environments, including woodlands and clinical origins [84,85], owing to the fact that clinical isolates show strong potency for invasive and pseudohyphal growth [85,86,87]. Yet, several questions remain unanswered on the structure–function of Flo11p, including the role of the number or the nature of the tandem repeats in adhesion and biofilm formation, the importance of the glycosylation state on Flo11p function, as well as to clarify how this protein can be retained on the cell wall while its GPI-anchoring site is removed.

Like *C. albicans* adhesins, the *S. cerevisiae* Flo11 flocculins can make nanodomains under the action of shear force, and we showed that this property depends on the presence of a threshold number of amyloid-forming sequences. In addition, it was shown that Flo11p mediates cell–cell adhesion by homotypic protein–protein interactions characterized by a strong adhesion force of more than 10 nN between two interacting cells [45]. Further experiments using e.g., AFM-based single-cell force spectroscopy (SCFS) or fluid force microscopy (FluidFM) [68] should be conducted to support the model that the formation of nanodomains (and thus cis-interaction between Flo11p on a same cell) would strengthen these adhesion forces. Nevertheless, these adhesion characteristics, common to those of *C. albicans* adhesins and complemented by the filamentous phenotype of Flo11p, make this flocculin a good model to study the mechanisms of cell–cell adhesion, cell–surface adhesion, and biofilm formation, which form the initial events leading to fungal infection by pathogenic yeasts like *C*. *albicans* and *C. glabrata.*

## Figures and Tables

**Table 1:** Predicted β-aggregating sequence (>30%) in Flo proteins from *Saccharomyces cerevisiae* S288c strain according to the predictor TANGO software.

	Flo1p			Flo5p			Flo9p			Flo10p			Flo11p		
Domain	AA Position	Motif	β-Aggregate (%)	AA Position	Motif	β-Aggregate (%)	AA Position	Motif	β-Aggregate (%)	AA Position	Motif	β-Aggregate (%)	AA Position	Motif	Mean β-Aggregate (%)
**A**	7–21	YMFLAVFTLLALTSV	82.2	9–22	IFVILAFLALINVA	82	6–22	YYCLLLAIVLLGLTNVV	77.6	7–23	YIFLTGLFLLSVANVAL	81.7	5–16	FLLAYVLSLLF	96
**B**	308–312	TVIVI	87.9	207–215	TVYMYAGYY	30.5	118–122	IIAYW	72						
	353–357	TIIVI	87.3	308–312	TVIVI	73.2	207–215	TVYMYAGFY	50.9						
	398–402	TIIVI	87.3	353–357	TVIVI	87.8	308–312	TVIVI	87.9						
	443–447	TIIVI	87.3	398–402	TVIVI	87.8	353–357	TIIVI	87.3						
	488–492	TIIVI	87.3	443–447	TVIVI	87.8	398–402	TIIVI	87.3						
	533–537	TIIVI	87.2	488–492	TVIVI	87.8	443–447	TIIVI	87.3						
	578–562	TIIVI	87.2	533–537	TVIVI	87.9	498–492	TIIVI	87.3						
	623–627	TIIVI	87.2	578–582	TVIVI	87.9	533–537	TIIVI	87.2						
	667–672	TIIVI	87.2	623–627	TVIVI	87.9	578–582	TIIVI	87.2						
	713–717	TIIVI	87.2	783–788	TLVTVT	31.7	623–627	TIIVI	87.2						
	758–762	TVIVI	87.8	802–811	AIVSTATVTV	45.2	668–672	TIIVI	87.2						
	803–807	TVIVI	87.8	855–859	TVVTI	37.2	713–717	TIIVI	87.2						
	848–852	TVIVI	87.8				758–762	TVIVI	87.8						
	893–897	TVIVI	87.8				803–807	TVIVI	87.8						
	938–942	TVIVI	87.8				848–852	TVIIV	87.,7						
	983–987	TVIVI	87.8				1021–1026	TLVTVT	31.9						
**C**	1028–1032	TVIVI	87.9				1040–1049	AIVSTATVTV	42.6						
	1073–1077	TVIVV	88.5				1093–1097	TVVTI	37.4						
	1234–1239	TLVTVT	31.7							1028–1035	VVTVYSTW	48.5	1033–1042	VTTVVSTTVV	75.8
	1254–1262	IVSTATVTV	45.9							1115–1119	VLISV	47	1056–1061	ITTTFV	56
	1299–1303	TVVTI	37.2	906–911	TLVTVT	36.1	1144–1149	TLVTVT	37.7	1157–1168	ISIFIASLLLAI	89.8	1133–1144	TLVTTAVTTTVV	84.8
	1350–1355	TLVTVT	36	1063–1074	LSVFIASLLLAI	87	1175–1182	VVTVYSTW	82.6				1356–1362	FMWLLLA	85.3
	1525–1536	LSVFIASLLLAI	87				1310–1321	LSVFIASLLLAI	87						

**Table 2:** Intragenic repeats in the middle region (B-domain) of *FLO11* gene from various *Saccharomyces cerevisiae* strains.

Strain	ORF (bp)	Repetition Length (TR)	Score	Count	Repetition Start (nt. seq.)	Repetition Stop (nt. seq.)	Repetition Conservation (%)	Repeated Sequence (Consensus)
S288c	4104	63	304	24	688	2388	60.8	tctactacagcaaccacttcaaccaccgcaactactgcaaccacttctactactgaaaccact
		33	55	8	2832	3095	66.7	ctctgcatgaacaaccactaccactacaactac
		45	48	3	2429	2563	84.4	caaccccatcaagctctagcactgaaagctcttctgctccagtat
		72	24	4	3099	3386	66.7	aactacagttttctccccaaacactgttactactacggtttcttctacaactacaactggtgcagacactac
Σ1278b	3633	81	438	13	767	1819	74.6	caaccagctctaccactgaaagctcttctgctccagctccaactccaaccagctctaccactgaaagctcttctgctccag
		45	94	5	1923	2147	80.9	cactgaaagctcttctgctccagtaccaactccatccagctctag
		45	46	3	2158	2292	83.7	ccagtaccaactccatccagctctagcactgaaagctcctctgct
		45	29	2	335	424	91.1	gttgcgacgaaaatacctatttgattgacaacccaactgatttca
133d	4890	81	1708	49	827	4795	72.5	cttcttctgctccagttacttcttctactactgaatcttcttctgctccagctcctactccttcttcttctactactgaat
L69	5166	63	323	30	658	2547	60.2	acttcatctaccgctactactgcaaccacttctactactgcaaccacttctactactgcaaca
		45	95	4	2646	2825	88.9	accagctccaactccatccagctctactactgaaagctcttctgc
		45	71	4	2958	3137	82.2	atccagctctaccactgaaagctcttctgctccagtatcaacccc
		12	41	8	2836	2955	73.3	agctctactgctcca
